# Identification of a Plasma Four-microRNA Panel as Potential Noninvasive Biomarker for Osteosarcoma

**DOI:** 10.1371/journal.pone.0121499

**Published:** 2015-03-16

**Authors:** Feng Lian, Yong Cui, Chenliang Zhou, Kewei Gao, Liwen Wu

**Affiliations:** Department of Orthopedics, The Fourth Affiliated Hospital of Harbin Medical University, Harbin, 150001, Heilongjiang, China; East Carolina University, UNITED STATES

## Abstract

**Background:**

Circulating microRNAs (miRNAs) are emerging as promising biomarkers for human cancer. Osteosarcoma is the most common human primary malignant bone tumor in children and young adults. The objective of this study was to investigate whether circulating miRNAs in plasma could be a useful biomarker for detecting osteosarcoma and monitoring tumor removal dynamics.

**Methods:**

Plasma samples were obtained from 90 patients before surgery, 50 patients after one month of surgery, and 90 healthy individuals. The study was divided into three steps: First, initial screening of the profiles of circulating miRNAs in pooled plasma samples from healthy controls and pre-operative osteosarcoma patients using a TaqMan low density array (TLDA). Second, evaluation of miRNA concentration in individual plasma samples from 90 pre-operative osteosarcoma patients and 90 healthy controls by a quantitative real time PCR (qRT-PCR) assay. Third, evaluation of miRNA concentration in paired plasma samples from 50 pre- and post-operative osteosarcoma patients by qRT-PCR assay.

**Results:**

Four plasma miRNAs including miR-195-5p, miR-199a-3p, miR-320a, and miR-374a-5p were significantly increased in the osteosarcoma patients. Receiver operating characteristics curve analysis of the combined populations demonstrated that the four-miRNA signature could discriminate cases from controls with an area under the curve of 0.9608 (95% CI 0.9307-0.9912). These 4 miRNAs were markedly decreased in the plasma after operation. In addition, circulating miR-195-5p and miR-199a-3p were correlated with metastasis status, while miR-199a-3p and miR-320a were correlated with histological subtype.

**Conclusions:**

Our data suggest that altered levels of circulating miRNAs might have great potential to serve as novel, non-invasive biomarkers for osteosarcoma.

## Introduction

Osteosarcoma is the most common primary malignant bone tumor with high morbidity in young adults and adolescents [1]. The development of multiple therapeutic strategies for osteosarcoma including wide tumor excision, adjuvant chemotherapy and radiotherapy has significantly improved the prognosis of patients with malignancy [2]. Despite extensive advancements in diagnostic methods and surgical techniques in recent years, the 5-year survival rate of osteosarcoma patients remains at 60–70% [3]. Therefore, discovery of sensitive and specific minimally invasive biomarkers that can be used to detect osteosarcoma at an early stage is one of the most important challenges in the management of osteosarcoma.

MicroRNAs (miRNAs) are short, endogenous, non-coding RNAs that regulate gene expression at the posttranscriptional level by binding to the 3’-untranslated regions (3’-UTRs) of their target mRNAs [4–5]. miRNAs regulate a variety of biological processes, including cell proliferation, differentiation, migration, metabolism, and apoptosis [6]. Interestingly, miRNAs have recently been shown to circulate in plasma, serum and other body fluids in a remarkably stable form [7]. More importantly, many studies have demonstrated that circulating miRNAs may be useful as biomarkers for diseases [7–8]. Liu et al. established a three-plasma miRNA signature as novel biomarkers for osteosarcoma [9]. Tian et al. found that plasma miR-34b was causally associated with osteosarcoma risk and related with its metastatic status [10]. Yuan et al. found that serum miR-21 was significantly correlated with advanced Enneking stage and chemotherapeutic resistance, and was an independent unfavorable prognostic factor for patients with osteosarcoma [11]. However, these studies have their own shortcomings, either only focused on individual miRNAs or just selected target miRNAs from literature, or not studied the dynamic change of miRNAs before and after surgery. In this study, we employed a high-throughput TaqMan low density array (TLDA), followed by a TaqMan probe-based stem-loop quantitative real time PCR (qRT-PCR) assay, to systematically and extensively investigate the plasma miRNA expression profiles in osteosarcoma.

## Materials and Methods

Patients with primary osteosarcoma, treated in the Department of Orthopedics, The Fourth Affiliated Hospital of Harbin Medical University between January 2007 and December 2013, were enrolled in this study. Inclusion criteria included: (a) clinical and histological diagnosis of primary osteosarcoma; and (b) first diagnosis without prior treatment. Plasma samples from healthy age- and sex-matched volunteers from the local population were used as controls. They underwent medical examinations and did not have any orthopedic disease or other cancerous disease. The pre-operative blood samples were obtained from 90 patients prior to surgery and/or chemotherapy. Paired plasma samples before surgery and one month after surgery were collected from 50 patients. All extracted plasma samples were stored in phased liquid nitrogen. To minimize the effect of freeze-thaw on circulating miRNAs, we only used plasma samples which had not been previously thawed. This study was approved by the Institutional Review Board of The Fourth Affiliated Hospital of Harbin Medical University, and each participant provided signed informed consent. The clinical and demographic characteristics of the patients and healthy controls are summarized in Table 1.

**Table 1:** Summary of the clinical and pathological characteristics of osteosarcoma and healthy control plasma samples.

Variable	Osteosarcoma (n = 90)		Control (n = 90)		p-value
	No.	%	No.	%	
**Average age (years)**	15.8 ± 8.1		16.2 ± 6.4		0.634 ^a^
**Age (years)**					0.881 ^b^
≤ 16	48	53.3	46	51.1	
> 16	42	46.7	44	48.9	
**Sex**					0.881 ^b^
Male	43	47.8	44	50.0	
Female	47	52.2	46	50.0	
**Tumor location**					
Femur	42	46.7			
Tibia/fibula	33	36.7			
Arm	8	8.9			
Central	7	7.7			
**Metastasis at diagnosis**					
Absent	72	80.0			
Present	18	20.0			
**Subtype**					
Osteoblastic	65	72.2			
Chondroblastic	25	27.8			

^a^ Student's t-test.

^b^ Two-sided λ^2^ test.

### Analysis of Plasma miRNA Profile by TLDA

The plasma samples from 10 healthy controls and 10 pre-operative osteosarcoma patients were pooled. Total RNA was isolated from each pool of plasma samples using the mirVana miRNA Isolation Kit (Applied Biosystems, Foster City, CA, USA) according to the manufacturer’s instructions. For cDNA synthesis, 30 ng of total RNA was subjected to reverse transcription using a TaqMan microRNA Reverse Transcription Kit (#4366596; Applied Biosystems) and Megaplex RT primers (Human Pool A + B; Applied Biosystems) following the manufacturer’s protocol, allowing simultaneous reverse transcription of 739 mature human miRNAs to generate a miRNA cDNA library corresponding to each plasma sample. Reverse transcription was performed with the following cycling conditions: 40 cycles at 16°C for 2 min, 42°C for 1 min and 50°C for 1 s followed by a final step of 80°C for 5 min to inactivate reverse transcriptase. Thereafter, to generate enough miRNA cDNA template for the following real-time PCR, the cDNA libraries were pre-amplified using Megaplex PreAmp primer (Humam Pool A + B; Applied Biosystems) and PreAmp Master Mix (#4384266; Applied Biosystems) following the manufacturer’s instructions. The PreAmp primer pool used here consisted of forward primers specific for each of the 739 human miRNAs and a universal reverse primer. The pre-amplification cycling conditions were as follows: 95°C for 10 min, 55°C for 2 min, 72°C for 2 min followed by 12 cycles at 95°C for 30 s and 60°C for 4 min; the samples were then held at 99.9°C for 10 min. After the preamplification step, the products were diluted with RNase-free water, combined with TaqMan gene expression Master Mix and then loaded into TaqMan Human MicroRNA Array A + B (Applied Biosystems), which are two 384-well formatted plates and real-time PCR-based microfluidic cards with embedded TaqMan primers and probes in each well for the 739 different mature human miRNAs. Real-time PCR was performed on an ABI PRISM 7900HT sequence detection system (Applied Biosystems) with the following cycling conditions: 50°C for 2 min, 94.5°C for 10 min followed by 40 cycles at 95°C for 30 s and 59.7°C for 1 min. The Ct (cycle threshold) was automatically given by SDS 2.3 software (Applied Biosystems) and is defined as the fractional cycle number at which the fluorescence passes the fixed threshold. U6 embedded in the TaqMan Human MicroRNA Arrays was used as an internal control. The relative expression levels of miRNAs were calculated using the comparative ΔΔCt method. The microarray data has been deposited in NCBI Gene Expression Omnibus (GEO) database under accession number GSE64915.

### RNA Isolation and qRT-PCR

Total RNA was isolated from 200 μL of human plasma and performed according to the manufacturer’s protocol of the mirVana PARIS miRNA Isolation Kit (Ambion 1556, Austin, TX) with the modification that each plasma sample was treated twice with the acid-phenol chloroform provided in the kit. The TaqMan microRNA assay (Applied Biosystems) was performed using a TaqMan PCR kit with the Applied Biosystems 7500 Sequence Detection System according to the instructions. Briefly, the reverse transcription reaction was performed in 20 μL mixture containing 3 μL of RNA extracted from the plasma, 1.5 μL of 10 mM dNTPs, 1.0 μL of AMV reverse transcriptase (TaKaRa, Dalian, China), 1.5 μL of a stem-loop RT primer (Applied Biosystems), 2.5 μL of 5 × reverse transcription buffer and 5.0 μL of DEPC-treated water. For synthesis of cDNA, the reaction mixture was incubated at 16°C for 30 min, 42°C for 70 min, 85°C for 10 min, and then held at 4°C. Real-time PCR was then performed with 1 cycle of 95°C for 10 min, followed by 40 cycles of 95°C for 30 sec and 60°C for 1.5 min. All reactions, including controls containing no template RNA, were performed in triplicate. The expression data for the miRNA were acquired and analyzed using the ABI PRISM 7500 Sequence Detection System and 7500 Software v2.0.1 (Applied Biosystems). The resulting Ct values were determined using fixed threshold settings. Due to the lack of a consensus housekeeping miRNA for qRT-PCR analysis of plasma miRNA, we selected a normalization method utilizing the comparison of the miRNA concentration to the plasma volume, as was described in previous report [12]. To calculate the absolute expression levels of the target miRNAs in plasma, a series of synthetic Caenorhabditis elegans miRNA cel-miR-39 (RNA oligonucleotides synthesized by Qiagen) of known concentrations (from 1 fM to 10^5^ fM) were also reverse-transcribed and amplified. The absolute amount of each miRNA was then calculated by referring to the standard curve.

### Statistical Analysis

The data were presented as the means ± SD. Student’s t-test or the two-sided χ^2^ test was used to compare the differences in the plasma miRNA concentrations between the two groups. Comparisons between more than two groups were performed using a one-way analysis of variance (ANOVA), and the differences between the groups were subsequently determined by the Fisher LSD test when appropriate. A p-value of < 0.05 was considered statistically significant. Risk score analysis was performed to evaluate the associations between the expression levels of the plasma miRNAs and osteosarcoma. The risk score of each miRNA, denoted as *s*, was set to 1 if the expression level was greater than the upper 95% reference interval for the corresponding miRNA level in the controls; otherwise, it was set to 0. A risk score function (RSF) to predict osteosarcoma risk was defined according to a linear combination of the expression level for each miRNA. For example, the RSF for sample *i* using the information from 4 miRNAs was $rsf_{i}=\sum_{j=1}^{4}W_{j}\cdot s_{ij}$. In the above equation, *s*_*ij*_ is the risk score for miRNA *j* on sample *i*, and *W*_*j*_ is the weight of the risk score of miRNA *j*. To determine the *Ws*, 4 univariate logistic regression models were fit using the disease status with each of the risk scores. The regression coefficient of each risk score was used as the weight to indicate the contribution of each miRNA to the RSF. Frequency tables and ROC curves were then used to evaluate the diagnostic effects of the profiling and to find the appropriate cutoff point.

## Results

### Characteristics of the Study Population

The distributions of selected characteristics of study subjects were shown in Table 1. The mean age of the osteosarcoma subjects was 15.8 ± 8.1 years old. Of 90 osteosarcoma patients, 43 (47.8%) patients were males, 47 (52.2%) patients were females, 42 (46.7%) patients showed tumor location at femur, 33 (36.7%) patients at tibia/fibula, 8 (8.9%) patients at arm, 7 (7.7%) patients at central, 18 (20.0%) patients showed metastasis at diagnosis, 65 (72.2%) patients were osteoblastic subtype of osteosarcoma, 25 (27.8%) patients were chondroblastic subtype of osteosarcoma.

### Selection of Candidate Plasma miRNAs for Osteosarcoma

**Fig 1:**
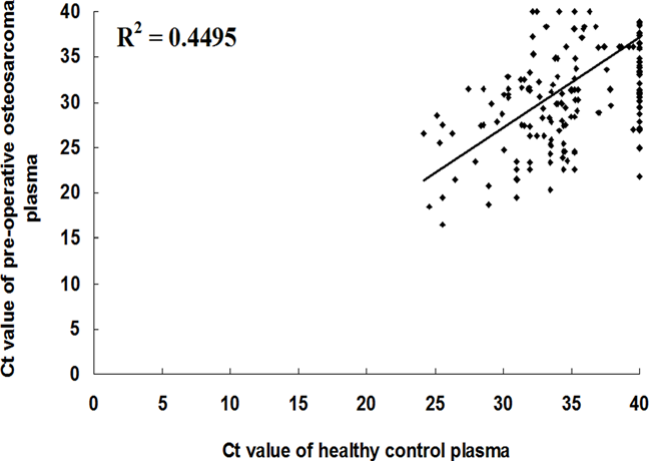
Analysis of plasma miRNA expression profiles by TLDA. Correlation and scatter plot analysis of miRNAs expression levels in pooled plasma samples from healthy controls and pre-operative osteosarcoma patients (each pooled from 10 individuals).

To select candidate plasma miRNAs for osteosarcoma detection and monitoring, we employed TLDA technique to screen expression levels of 739 miRNAs in pooled plasma samples from healthy controls and pre-operative osteosarcoma patients (each pooled from 10 individuals). The results revealed that plasma miRNA expression profiles varied between pre-operative osteosarcomas and healthy controls. Correlation and scatter plot analyses revealed that the correlation coefficients were low; the R^2^ value was 0.4495 between the pre-operative osteosarcoma patients and the healthy controls (Fig. 1). We next narrowed down the list of miRNAs to be used in osteosarcoma detection and monitoring. The following criteria were used to select the miRNA for further analysis: 1) only upregulated miRNAs were included, and 2) a fold change > 200. Considering that the plasma levels of miRNAs are upregulated in patients as compared to normal donors in most cancer and other diseases [13], we only studied those upregulated miRNAs in this study. Consequently, 55 miRNAs that met the inclusion criteria were chosen for further qRT-PCR analysis (S1 Table).

### Validation of Differentially Expressed Plasma miRNAs by Individual qRT-PCR Analysis

**Fig 2:**
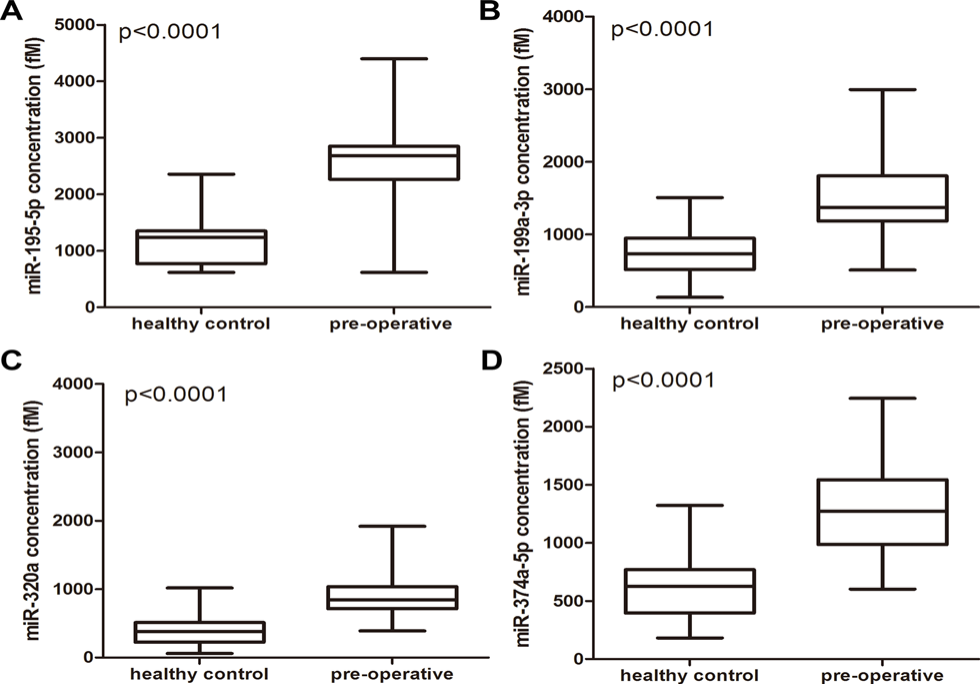
The expression levels of the 4 miRNAs in the plasma of 90 osteosarcoma patients and 90 healthy control individuals, as determined by qRT-PCR analysis.

Next, the 55 candidate miRNAs were individually assayed by qRT-PCR in 90 pre-operative osteosarcoma samples and 90 healthy controls (the individuals used to create the pools were excluded) to validate their differential expression and to investigate whether anyone could be used as a biomarker for osteosarcoma diagnosis. The miRNAs were considered to be significantly differentially expressed only when they exhibited a mean change ≥ 2.0-fold, a p-value < 0.05. Our analysis ultimately generated a list of 4 miRNAs that were differentially expressed in the pre-operative osteosarcoma plasma samples compared to the healthy controls. The 4 miRNAs, including miR-195–5p, miR-199a-3p, miR-320a, and miR-374a-5p, were shown to be upregulated by a factor greater than two-fold. The differential expression of the 4 miRNAs in the 90 pre-operative osteosarcoma plasma samples compared to the 90 healthy controls is shown in Fig. 2.

### Selected miRNAs as Potential Biomarkers for Osteosarcoma Diagnosis

**Fig 3:**
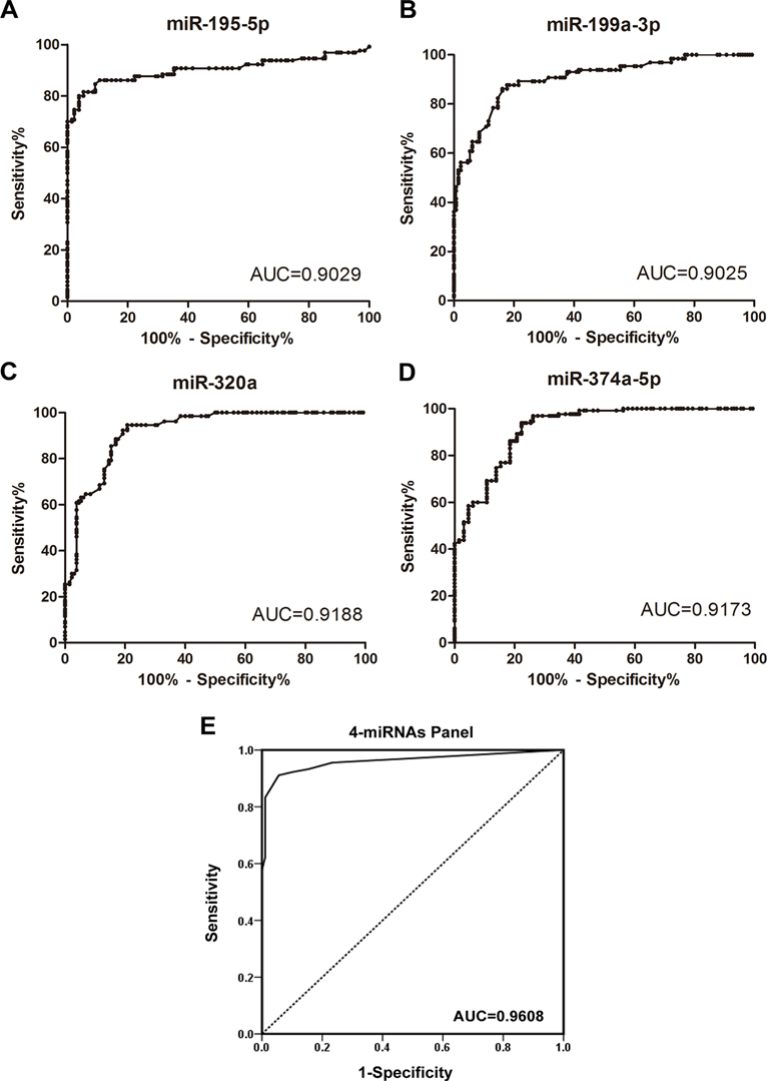
Selected miRNAs as potential biomarkers for osteosarcoma diagnosis. The ROC curves indicate the ability of the plasma levels of the 4 miRNAs to differentiate the osteosarcoma patients from the control subjects (A-D). (E) The ROC curves for the 4-miRNA profile to distinguish the osteosarcoma samples from the control samples.

ROC curve analysis was performed to evaluate the diagnostic potential of the 4 miRNAs in the plasma for the detection of osteosarcomas. The discriminatory power between the tumor and control samples is depicted by the areas under the curves (AUCs). The AUC was 0.9029 for miR-195–5p (95% confidence interval [CI], 0.8602–0.9456) (Fig. 3A), 0.9025 for miR-199a-3p (95% confidence interval [CI], 0.8658–0.9392) (Fig. 3B), 0.9188 for miR-320a (95% confidence interval [CI], 0.8857–0.9519) (Fig. 3C), and 0.9173 for miR-374a-5p (95% confidence interval [CI], 0.8855–0.9492) (Fig. 3D). To further evaluate the diagnostic value of this miRNA profiling system, we used a risk score formula to calculate the risk score for the patient samples and control samples. The samples were ranked according to their risk score and then divided into a high-risk group, which represented the predicted osteosarcoma cases, and a low-risk group, which represented the predicted control individuals. The frequency table and the ROC curves were then used to evaluate the diagnostic effects of the 4-miRNA panel. The AUC for the combined 4 miRNAs was 0.608 (95% CI, 0.9306–0.9910) for the osteosarcomas and controls (Fig. 3E). With an optimal cutoff value (RSF = 3.779) at which the sum of the sensitivity and specificity was maximal, the sensitivity was 91.1%, and the specificity was 94.4% for osteosarcoma. These results demonstrate that the combined analysis of these 4 miRNAs could differentiate osteosarcoma patients from healthy controls.

### Selected miRNAs as Potential Biomarkers for Evaluating Tumor Removal

**Fig 4:**
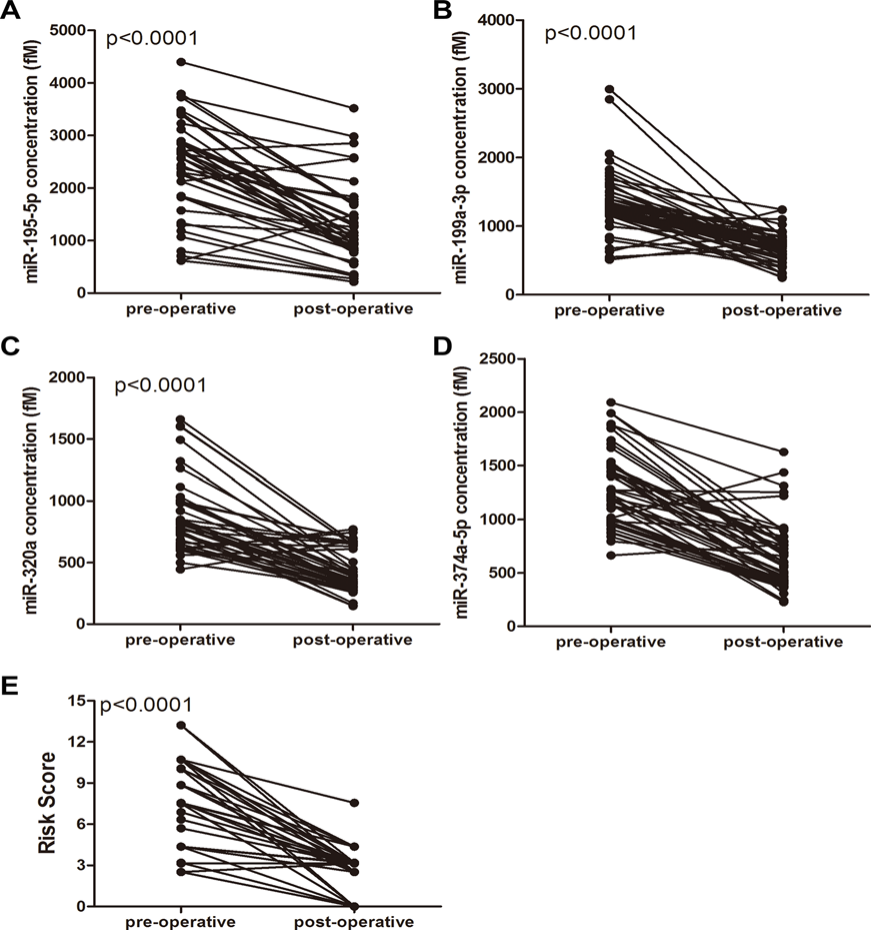
The dynamic change of plasma miRNAs before and after surgery. The expression levels of the 4 miRNAs in the patients before and after surgery (A-D). Each point represents the mean of the triplicate samples. (E) The risk score of the 4 miRNAs in the osteosarcoma patients before and after surgery.

Subsequently, the expression levels of the 4 miRNAs were analyzed in the paired pre- and post-operative osteosarcoma plasma samples from 50 patients who underwent surgical removal of the tumors. The levels of the 4 miRNAs were significantly reduced in the post-operative samples compared to the levels in the pre-operative samples (Figs. 4A–4D). To further evaluate the monitoring value of this miRNA profiling system, a paired t-test was used to compare the pre- and post-operative risk score. The post-operative risk score was significantly lower than the pre-operative risk score (p < 0.0001) (Fig. 4E). These findings imply that these plasma miRNAs may reflect tumor dynamics and are available as new biomarkers to evaluate tumor removal.

### Relationships between Plasma miRNAs and Clinical Factors

**Fig 5:**
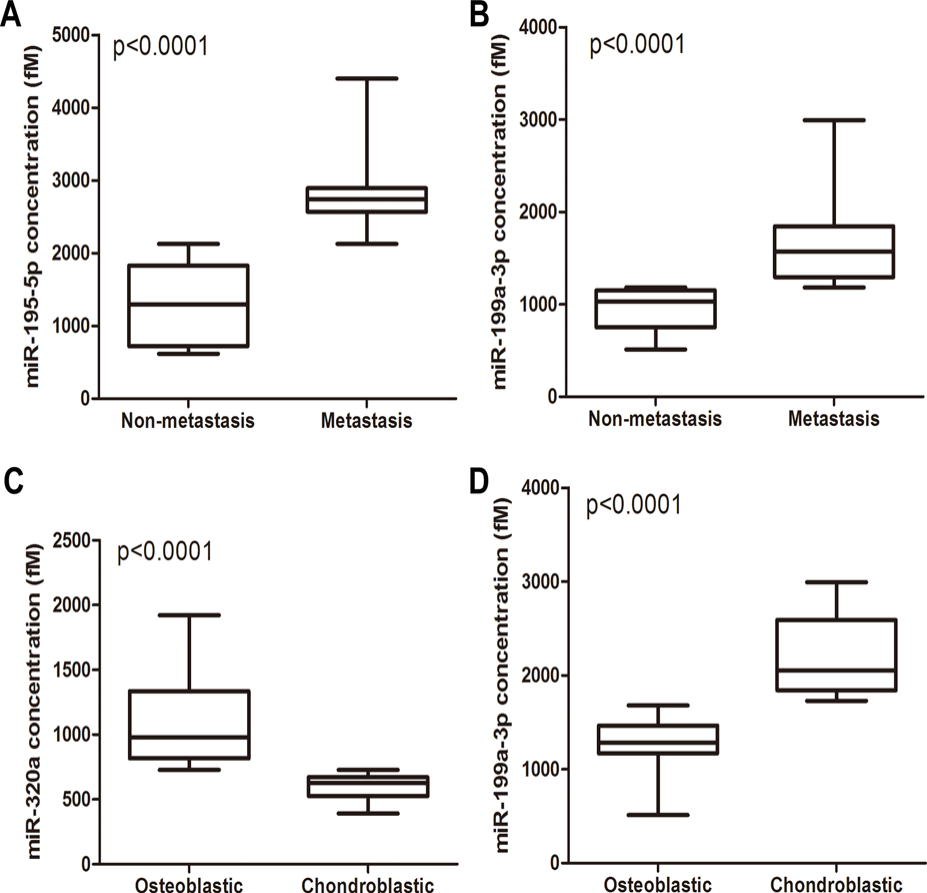
Relationships between the expression levels of plasma miRNAs and clinical presentations of osteosarcoma patients. (A) The correlation between the expression levels of miR-195–5p and metastasis status. (B) The correlation between the expression levels of miR-199a-3p and metastasis status. (C) The correlation between the expression levels of miR-320a and histological subtypes. (D) The correlation between the expression levels of miR-199a-3p and histological subtypes.

Correlations between circulating miRNA levels and the clinical presentations were analyzed in the total 90 osteosarcoma patients. Plasma levels of miR-195–5p and miR-199a-3p were significantly increased in the metastatic patients compared with the non-metastatic ones (Fig. 5A-5B). No significant difference in levels of miR-320a and miR-374a-5p were observed between the two groups. Furthermore, the plasma levels of miR-195–5p and miR-199a-3p were analyzed in the 50 patients without or with metastasis who underwent surgical removal of the tumors. As shown in S1 Fig., both miR-195–5p and miR-199a-3p were significantly decreased in the patients without and with metastasis after tumor removal. miR-320a showed significantly higher levels in the osteoblastic patients than the chondroblastic ones (Fig. 5C), while circulating levels of miR-199a-3p was significantly decreased in patients with osteoblastic subtype (Fig. 5D).

## Discussion

In this study, we found that the 4-miRNAs panel had a high potential of distinguishing the osteosarcoma patients from the normal controls. Moreover, the levels of all the selected miRNAs markedly decreased after the surgical removal of the primary tumors, suggesting the clinical potential of the identified plasma miRNAs in estimating the effect of surgery for osteosarcomas. In addition, levels of the circulating miRNAs were correlated with tumor metastatic status and histological subtypes.

While the molecular basis of osteosarcoma has received considerable attention during the past decade, it remains difficult to detect and monitor osteosarcoma at early stage [14–15]. Recent studies have revealed that circulating miRNAs are noninvasive diagnostic or prognostic biomarkers for various kinds of diseases, especially in the field of cancer [8, 16]. The discovery that plasma miRNAs can serve as potential cancer biomarkers overcomes the problem of collecting tissue samples through invasive procedures such as biopsy or surgery. Plasma samples are easily acquired in a relatively noninvasive manner, and isolated miRNAs are readily detected by qRT-PCR, a widely used clinical and laboratory technique. Since circulating miRNAs are highly correlated with each other, miRNA signatures rather than individual miRNAs may be more reliable biomarkers for diseases [17]. Therefore, the determination of the plasma miRNA profiles in osteosarcoma patients is quite meaningful, which would allow the comprehensive analysis of diseases in a less invasive manner and in an early stage. This new approach will definitely have a huge impact on the management of osteosarcoma in the future.

As we known, miRNAs play crucial roles as oncogenes or tumor suppressors, and their deregulation is involved in multiple processes including cell proliferation, apoptosis, cell-cycle regulation and invasion in various diseases [4, 18]. Metastasis status at initial presentation and histological type are two patient-related factors which can predict the survival of osteosarcoma [19]. In this study, miR-195–5p and miR-199a-3p were correlated with metastatic status, and miR-320a and miR-199a-3p were correlated with osteoblastic subtype. It has been reported that miR-195–5p could inhibit osteosarcoma cell migration and invasion through targeting FASN (fatty acid synthase) [20]. miR-199a-3p could inhibit osteosarcoma cell growth, migration, and induce the apoptosis via p53 signaling pathway [21–22]. Although the roles of miR-320a and miR-374–5p have been reported in many different types of cancer, there are no direct reports of these miRNAs in osteosarcoma to date. The underlying mechanism and their biological relevance in osteosarcoma still needs to be determined in future studies.

In conclusion, our study supports the use of plasma miRNAs as non-invasive biomarkers for the diagnosis and prognosis of osteosarcomas. This observation will trigger further research to elucidate the functional effects of these miRNAs, which will improve our knowledge regarding the role that these novel biomarkers play in carcinogenesis and will expose their true potential as therapeutic agents.

## Supporting Information

S1 Fig The expression levels of plasma miRNAs in osteosarcoma patients without and with metastasis before and after surgery. (A) The expression levels of miR-195–5p in patients without metastasis before and after surgery. (B) The expression levels of miR-195–5p in patients with metastasis before and after surgery. (C) The expression levels of miR-199a-3p in patients without metastasis before and after surgery. (D) The expression levels of miR-199a-3p in patients with metastasis before and after surgery.
(TIF)

S1 Table Differentially expressed miRNAs in pre-operative osteosarcoma plasma samples compared to healthy control plasma samples determined by TLDA. (XLS)
