# Supplementary figures and images for: Identification of a Plasma Four-microRNA Panel as Potential Noninvasive Biomarker for Osteosarcoma
Source: PLoS One. 2015 Mar 16;10(3):e0121499. doi: 10.1371/journal.pone.0121499 (PMC4361617; doi:10.1371/journal.pone.0121499)

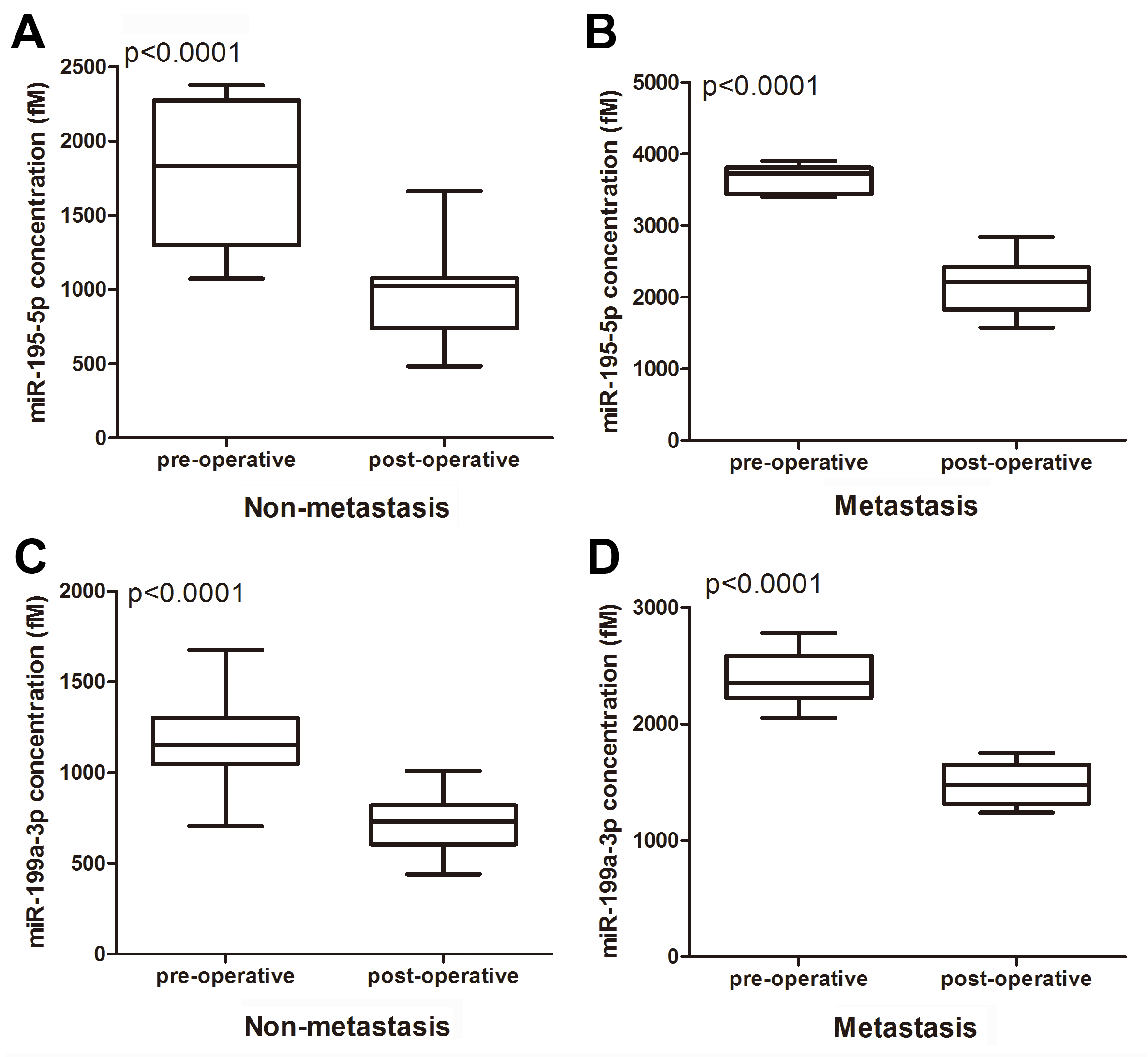

Supplement: S1 Fig — (A) The expression levels of miR-195–5p in patients without metastasis before and after surgery. (B) The expression levels of miR-195–5p in patients with metastasis before and after surgery. (C) The expression levels of miR-199a-3p in patients without metastasis before and after surgery. (D) The expression levels of miR-199a-3p in patients with metastasis before and after surgery. (TIF) [file pone.0121499.s001.tif]
